# Applications of Biotechnology to the Craniofacial Complex: A Critical Review

**DOI:** 10.3390/bioengineering9110640

**Published:** 2022-11-03

**Authors:** Ioannis A. Tsolakis, Isidora Christopoulou, Erofili Papadopoulou, William Papaioannou, Konstantina-Eleni Alexiou, Ioannis Lyros, Aliki Rontogianni, Christina-Efthymia Souliou, Apostolos I. Tsolakis

**Affiliations:** 1 Department of Orthodontics, School of Dentistry, Aristotle University of Thessaloniki, 541 24 Thessaloniki, Greece; 2 Department of Orthodontics, School of Dentistry, National and Kapodistrian University of Athens, 106 79 Athens, Greece; 3 Department of Oral Medicine & Pathology and Hospital Dentistry, School of Dentistry, National and Kapodistrian University of Athens, 106 79 Athens, Greece; 4 Department of Preventive & Community Dentistry, School of Dentistry, National and Kapodistrian University of Athens, 106 79 Athens, Greece; 5 Department of Oral Diagnosis & Radiology, School of Dentistry, National and Kapodistrian University of Athens, 106 79 Athens, Greece; 6 Oral and Maxilla-Facial Surgeon, Department of Oral and Maxillofacial Surgery, Georgios Gennimatas Athens Hospital, 115 27 Athens, Greece; 7 Department of Orthodontics, Case Western Reserve University, Cleveland, OH 44106, USA

**Keywords:** biotechnology, bioengineering, scaffolds, stem cells, biomarkers, tissue regeneration

## Abstract

Background: Biotechnology shows a promising future in bridging the gap between biomedical basic sciences and clinical craniofacial practice. The purpose of the present review is to investigate the applications of biotechnology in the craniofacial complex. Methods: This critical review was conducted by using the following keywords in the search strategy: “biotechnology”, “bioengineering”, “craniofacial”, “stem cells”, “scaffolds”, “biomarkers”, and ”tissue regeneration”. The databases used for the electronic search were the Cochrane Library, Medline (PubMed), and Scopus. The search was conducted for studies published before June 2022. Results: The applications of biotechnology are numerous and provide clinicians with the great benefit of understanding the etiology of dentofacial deformities, as well as treating the defected areas. Research has been focused on craniofacial tissue regeneration with the use of stem cells and scaffolds, as well as in bioinformatics with the investigation of growth factors and biomarkers capable of providing evidence for craniofacial growth and development. This review presents the biotechnological opportunities in the fields related to the craniofacial complex and attempts to answer a series of questions that may be of interest to the reader. Conclusions: Biotechnology seems to offer a bright future ahead, improving and modernizing the clinical management of cranio-dento-facial diseases. Extensive research is needed as human studies on this subject are few and have controversial results.

## 1. Introduction

Biotechnology has entered the field of the cranio-dento-facial complex with new applications that modernize the clinical practice and close the gap between biomedical sciences and clinical practice. Biotechnological opportunities have the potential to greatly improve and modernize cranio-dento-facial orthopedic and surgical treatment significantly. The importance of biotechnology has enlarged as the result of three scientific domains that have experienced rapid development in recent years: molecular biology, computer modeling, and greater clinic-related experimentation. More specifically, biotechnology in craniofacial biology can be categorized under four broad domains: diagnosis, treatment rational, therapeutics, and monitoring. It encompasses stem cells, scaffolds, and genetic engineering. All these domains impact treatment and the regeneration of lost or injured structures. They also impact on orthodontics and dento-facial development. Specific biomarkers and sampling tests that provide clinicians with accurate and valid information will also be available in the near future via biotechnology, affecting orthodontic tooth movement and controlling or predicting craniofacial growth [1]. Given the breadth of applications and the great number of questions that the presently available biomedical tools and techniques raise, the use of biomarkers may provide the craniofacial field with a great opportunity to understand the etiology of dento-facial deformities, as well as to improve clinical practice with more personalized treatment plans.

Unfortunately, the availability of good biomedical evidence has not yet led to its adoption in clinical practice due to funding issues or limitations arising from several unsolved problems. Consequently, the problem raised is that biomedical evidence is not always translated into treatment, such that there is a gap between biotechnology and craniofacial practice. Specific strategies and approaches that are not commonly considered in efficacy studies are required to address this failing gap between biotechnology and craniofacial practice. The future seems to be promising with many different possibilities in everyday clinical practice. Since relevant information regarding biotechnology in craniofacial science has not yet been summarized in an evidence-based manner, the objective of this review is to investigate the available data and appraise the evidence on the use of biotechnology in the craniofacial complex [2].

There are currently few and inconclusive evaluations that look at multiple facets of this topic at once, with the exception of some clinical studies or reviews that have focused only on a single area of biotechnology in the craniofacial complex. For these reasons, we made the decision to complete a critical review using a standard structure, and we try to respond critically to a number of issues that the reader could find interesting.

This review was conducted by using the following keywords in the search strategy: “biotechnology”, “bioengineering”, “craniofacial”, “stem cells”, “scaffolds”, “biomarkers”, and ”tissue regeneration”. The databases used for the electronic search were the Cochrane Library, Medline (PubMed), Embase (via Ovid), CENTRAL, Google Scholar, and Scopus. The search was conducted for studies published before June 2022.

## 2. Relevant Sections

The use of biotechnology in orthodontics is applied in several ways, with different procedures and applications. Craniofacial tissue regeneration, stem cells, scaffolds with or without 3D printing, and biomarkers for orthodontic tooth movement or the prediction of craniofacial growth are the key parameters of biotechnology that are investigated in detail throughout this review. The biotechnological applications used for the regeneration of the cranio-dento-facial complex are summarized in Table 1.

### 2.1. Craniofacial Defects

The craniofacial complex is composed of a wide range of specialized tissues such as craniofacial bones, cartilages, muscles, ligaments, nerves, and blood arteries, and it is also composed of teeth, which are considered highly specialized structures. Together, these components support the craniofacial complex’s aesthetics, as well as numerous important tasks such as speaking and mastication. Craniofacial abnormalities are most frequently caused by congenital birth malformations, trauma, inflammation, and cancer procedures [3]. The need for an efficient, accurate, and aesthetically pleasing reconstruction is highlighted by the fact that craniofacial deformities can affect the structure and function of the jaws, as well as cause psychological problems. A lot of the problems faced by non-craniofacial tissue reconstructions are also problems faced by craniofacial tissue reconstructions. As a result, many theories and methods for general tissue engineering can also be used for the regeneration of craniofacial tissue [4].

Stem-cell-based bone tissue creation has been acknowledged as a promising alternative for bone reconstruction. Friedenstein’s team published the first findings indicating that fibroblast-like cells might develop into osteogenic cells, in addition to haematopoietic cells, as early as 1968 [5,6]. At that time, the cells were named “mechanocytes”. Similar studies have suggested that these cells are chondrogenic and adipogenic [5]. To date, these cells are commonly called mesenchymal stem cells (MSCs). MSC-mediated bone regeneration has been widely tested in several clinical trials, demonstrating that the local delivery of MSCs can enhance bone regeneration [7,8].

When discussing the difficulties in autologous bone for grafting craniofacial defects, the main obstacle is that it is only available in limited amounts. The field of bone-tissue engineering has been proposed as a realistic method to cure craniofacial deformities by mixing bioactive carriers, cells, and growth factors in order to overcome this and produce bone grafts of suitable complicated shapes. As previously indicated, MSC sources have demonstrated adequate osteogenic potential for bone regeneration and have been suggested as promising cell sources for cranial bone-tissue engineering [9]. More specifically, adult bone marrow stem cells are the most studied and are often used as stem cells for bone repair (BMSCs). In 2001, it was shown that mixing autologous sheep BMSCs with calcium alginate gel enhanced the healing of cranial lesions. [10]. Since then, MSCs from a variety of species, including mice, rats, and rabbits, have been confirmed as suitable cells for craniofacial bone repair [11,12,13].

Orthodontic and/or orthopedic treatment can be beneficial in achieving regeneration, as can stem cells and scaffolds. SCs from teeth can be acquired without further morbidity because the excision of primary teeth, permanent premolars, or wisdom teeth is a common clinical act in orthodontic treatment. The application of SCs in the treatment of dentofacial abnormalities and temporomandibular joint (TMJ) disorders, as well as their potential role in distraction osteogenesis (DO), have been reviewed in order to assess the uses of SCs in dentofacial orthopedics. Face reconstruction has a promising future due to distraction osteogenesis and the regeneration capabilities brought by stem cell therapy. In the distraction osteogenesis procedure, the injection of MSCs, before the onset of distraction, results in an increase in new bone volume in the distracted callus and in the bone mineral density (BMD) [14]. MSCs injection following distraction has revealed greater histological callus, new bone volume, and increased thickness of the new trabeculae, as well as a higher radiodensity of the distraction zone [15]. SCs from various sources, alone or in combination with genes and growth factors, can lead to an increase in bone volume and quality [14,16,17,18,19,20,21], bone mineral density [14,15,16,17,18], trabecular thickness [18,19], and biomechanical strength [22].

Several case reports and case series in the published literature have also reported results of MSCs usage in regenerating alveolar bone in clefts [20]. In one case, a composite scaffold made of demineralized bone material and calcium phosphate that was loaded with MSCs demonstrated 34.5% bone regeneration in the cleft area, whereas in the other, 25.6% of the bone was still present [21]. Approximately 50% fill of the bone defect was measured after placement of the scaffold, growth factor, and MSCs in the cleft area [23], whereas 79.1% bone regeneration was shown by another study [24]. SCs seem to present as a favorable option for bone regeneration in the oro-maxillofacial region with potential use in alveolar defect regeneration and reducing defect sizes by new bone formation [23,25,26], with less postoperative morbidity compared to autogenous bone grafting [27]. It also seems that teeth in the defect area tend to erupt in their proper position [28].

### 2.2. 3D Printing

**Figure 1:**
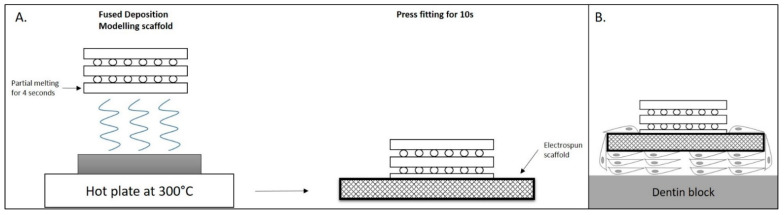
(**A**). Fabrication of the biphasic scaffold. (**B**). Biphasic scaffold assembled onto the dentin block.

Apart from stem cells, the 3D printing techniques designed to achieve functional organ regeneration are a promising tool, though they are still in their infancy [29]. Three-dimensional printed scaffolds, tissue analogs, and organs have been proposed as exciting alternatives to address some of the key challenges of regenerative medicine and dentistry [30,31]. This technique has the advantages of enabling the precise 3D positioning of cells and biomaterials while being customizable to patient-specific needs. Current 3D techniques for craniofacial regeneration are limited to bone and cartilage tissues [32,33]. Scaffolds, consisting of both soft and hard tissue components, have been used with success for periodontal regeneration [34], and their possible further applications are still under investigation. Three-dimensional printed biphasic scaffolds containing poly-(epsilon)-caprolactone and hydroxyapatite in the size and shape of teeth have been also tested [35]. During fabrication of the biphasic scaffold for periodontal regeneration use, the FDM component is held at a 1 cm distance from a hot plate, which results in the partial melting of the bottom layer. Before implantation, the tissue engineered constructs (TEC) are placed onto the dentin block and fixed using sutures. Afterward, it is ready to be implanted subcutaneously (Figure 1).

The craniofacial complex contains multiple types of highly integrated hard and soft tissues. Consequently, it would be of great benefit to regenerate hard and soft tissues simultaneously to achieve rapid functional recovery. It should be reported that the accurate integration of biological components and gradients for composite tissue engineering presents a significant barrier to the 3D printing process. When compared to alternative tissue-regeneration methods, 3D printing enables rather precise control over various tissues. The construction of 3D structures is based on the accumulation of 2D structures, and hence, 3D printing has its own set of limits [36,37].

### 2.3. Biomarkers

An interesting application of biotechnology-biomedicine in the field of orthodontics arises from the use of specific biomarkers. Inflammatory and molecular mechanisms underpinning bone and tissue remodeling in response to orthodontic force application may be detected by microRNAs (miRNAs), which are short, single-stranded, non-coding RNA molecules. Orthodontic pressures as mechanical stimuli are known to generate a variety of pro-inflammatory markers, which, in turn, triggers a cascade of cellular and metabolic processes that lead to bone remodeling in both mice and humans. These mediators are known to play an active role in osteoclastogenesis (e.g., interleukin (IL-1β), tumor necrosis factor (TNF)-α, receptor activator of nuclear kappa ligand (RANKL), etc.), as well as osteoblastogenesis (e.g., osteoprotegerin (OPG), IL-4, IL-10, etc.) [38,39,40]. Additionally, osteoclastic/osteoblastic differentiation linked to the production of several biomarkers in the paracrine system is coupled with the expression of various miRNAs at various observation times in orthodontic tooth movement (OTM) [41,42,43]. These biomarkers have created a new era in the field of orthodontics with promising future applications. Differential gene expression can affect orthodontic tooth movement by enhancing or decreasing it, depending on the proposed treatment plan. In the near future, the differential expression of several genes can be monitored in serum and bio-fluids (GCF and saliva) due to the ease in sampling and possible repeated evaluation. Through this procedure, precise and personalized orthodontic and dentofacial orthopedic treatments could become a reality. This may also be beneficial for diminishing treatment times and reducing the side effects of root resorption, caries risk, and gingival inflammatory conditions, as well as for predicting and monitoring craniofacial growth, which is a key issue in orthodontics [44].

### 2.4. TMJ Regeneration

Intra-articular positioning and/or structural abnormalities are the characteristics of TMJ disorders. The most prevalent type of TMJ problems is characterized by the displacement of the TMJ articular disc, which results in advancing osteoarthritis (OA). With the advent of stem cell-based therapies in recent years, advancements in the treatment of TMJ disorders have been made. Alternative approaches to treating disease symptoms and even replacing damaged tissue have been made possible via tissue engineering. Synovium-derived MSCs from adult MSCs outperformed chondrocytes in terms of proliferation and differentiation [45,46,47,48]. Distinct fibrocartilage formation is observed with the characteristic deposition of type I and II collagens formed after 4 weeks of in vivo implantation [49]. However, the efficacy of MSCs in cartilage regeneration is likely to be complicated by the abnormal mechanical loading and inflammation present in the defect area [50,51]. It is highly anticipated that the next-generation TMJ implants will be biological constructs fabricated using tissue engineering technology [51,52,53].

A wide variety of scaffolds have been synthesized from natural and/or synthetic polymers in the forms of sponges, fibrous meshes, and hydrogels [54,55,56]. The biomaterial scaffold plays a crucial role in the delivery of stem cells for TMJ repair by offering support and direction for cellular development and matrix deposition. To enable tissue remodeling and to aid in the formation of new tissue with the right anatomical shape and functional matrix anisotropy, an ideal scaffold should be biodegradable. A number of biomaterials, such as polylactic acid (PLA) [54], polyglycolic acid (PGA) [54,55], fibrin [49], chitosan [49], and poly glycerol sebacate (PGS) [57] have been used in TMJ bioengineering. Among these biomaterials, PGA is the most commonly used. However, PGA shows rapid degradation, leading to losses in structural and mechanical integrity over time.

Recently, it has been proposed that by regulating the amount of mesenchymal condensation during chondrogenesis, the degree of crosslinking and the matrix stiffness of the hyaluronic acid hydrogel scaffold could enable the differentiation of encapsulated MSCs into the formation of various types of cartilage tissues [58]. In the near future, extensive research will be conducted to incorporate specific bioactive ligands and growth factors into the scaffold, together with exogenous stimulation with mechanical forces, to enable chondrogenesis and osteogenesis [59].

### 2.5. Tooth Tissue Regeneration

In dentistry, stem cell-based therapy strategies can be an option since they can produce structural and functional outcomes that are physiologically superior. For the implantation of these therapies, a sufficient quantity of particular stem cell types is needed. Dental mesenchymal stem cells are simple to isolate and can be grown in vitro while still being stem cells. Dental mesenchymal stem cells have the ability to enhance pulp and periodontal regeneration, according to in vivo studies in both small and large animals, but they also have significant drawbacks and restrictions [60]. Dental pulp stem cells, periodontal ligament stem cells, dental apical papilla stem cells, human exfoliated deciduous teeth stem cells, dental follicle stem cells, dental epithelial stem cells, bone marrow mesenchymal stem cells, adipose-derived stem cells, embryonic stem cells, and induced pluripotent stem cells are all examples of stem cells used for tooth and periodontal regeneration (iPSCs) [61]. It should be highlighted that the fates and functions of stem cells after transplantation need to be further determined in future studies. Although tooth stem cell banking and clinical trials have been organized, their exact beneficial results for patients need to be closely monitored.

### 2.6. Polymers

#### 2.6.1. Natural Polymers

In dentistry, chitosan, collagen, hyaluronic acid, alginate, and albumin can be used to substitute an extracellular matrix of skeletal muscle, bone, or periodontium [62,63]. Hydrogels are safe and bioresorbable, but compared to cements, their mechanical properties are not sufficient and require improvement. As far as cements are concerned, there are two types: polymethyl methacrylate (PMMA) and calcium-phosphate cement (CPC). Unfortunately, bone cements are not resorbable, do not adhere to bone, and do not induce osteogenesis. Moreover, the reaction is exothermic, causing several problems. Another natural polymer useful in craniofacial regeneration is collagen, as mentioned above. Collagen membranes can improve bone regeneration and restoration. 

Even while natural polymers show good cell biocompatibility, their capacity to promote cell survival and function is currently being closely examined. Because natural polymers have limited mechanical strength and a rapid rate of degradation, it is common to combine them with other semi-synthetic or synthetic polymers, or ceramics, to slow the pace of breakdown. [64].

#### 2.6.2. Clear Overlay Appliances

Clear overlay appliances (COAs) are a recent trend in the field of orthodontics. The most popular methods of these are Invisalign^®®^ and ClearAligner^®®^. These splints, based on poly (ethylene terephthalate)-glycol (PETG), are a good alternative for classic brackets, especially due to their high esthetic appearance. The only disadvantages of this type of appliances are their low durability and weak antibacterial properties. To eliminate these drawbacks, multilayer films composed of chitosan and carboxymethyl cellulose have been recently proposed [65]. These additions enable the formation of a porous and rough film [65]. The purpose of those layers is to block the adhesion of bacteria and to improve the resistance and stability of PETG [65]. It remains to be seen whether aligner companies will adopt these techniques to enhance their products and become even more appealing to both patients and orthodontists [66].

## 3. Discussion

Biotechnology has made enormous steps in its applications in the field of orthodontics. However, all the available studies investigate the long-term survival and response of these new materials, with few data on their long-term stability [67]. There is also little evidence on the reproducibility of procedures involving stems cells and scaffolds. Moreover, a majority of the existing studies are animal studies, while human studies are mostly case reports or case series. It should be highlighted that at this point, which biomaterial is best for each case and how these materials are degraded over the course of time remains unknown. Consequently, more basic research and clinical trials are indispensable before any recommendations can be made with certitude. Furthermore, guidelines concerning the optimal time point to initiate such treatment procedures should be established. Regarding the repair of craniofacial defects with these new methods, one of the main obstacles in translating experimental observations into clinical practice is the relatively poor mechanistic understanding of stem-cell-mediated therapies [68].

One of the key issues that needs to be solved is the current lack of effective ways to control MSC destiny, particularly in the in vivo environment. Before stem cell-mediated therapies are used as the norm in regenerative medicine, we must have a greater grasp of how to maximize regenerative approaches. Preventing the ingrowth of connective tissue into the bone space is one of the key problems for successful bone healing in craniofacial regeneration, especially for large and complex cranial bone repair. To achieve this, the concept of guided tissue regeneration, which can be achieved by placing an inert membrane barrier over the defect to block the ingrowth of connective tissue, has been widely used for successful craniofacial reconstruction. However, the elevated cost and the increased difficulty currently limit these procedures in clinical practice. The use of 3D technology has minimized potential errors, up to a point, and has increased the accuracy of the procedures. MSC isolation and selection is another severe challenge that needs to be overcome [69]. One of the most promising solutions is to create 3D culture conditions that resemble the in vivo stem cell niche [70,71]. Three-dimensional cell cultures in bioreactors, combined with gene therapy or growth factors, have also been promising for increasing the survival of bone substitutes (Table 2).

As far as OTM is concerned, although salivary miRNAs have not yet been explored in OTM, they are being sufficiently investigated in the early screening of pathological conditions such as chronic periodontitis, OSCC, Sjogren syndrome, etc., and they have also been established in developmental defects such as CLP. Even though various biomarkers have been identified in oral fluids at the biochemical level, the evidence is still sparse. Hence, a critical evaluation of existing literature is required to identify the most potent miRNA biomarkers in OTM, their time-dependent alteration, and their effect on gene expression profiles and protein–protein interactions (PPI) to understand the biology of hard and soft tissue alterations and remodeling. As far as the limitations of existing studies are concerned, differences in sex, ethnicity, and age (adults and juveniles) need to be addressed to correctly comprehend the differences in the bone and tissue remodeling associated with OTM. A larger patient sample is also preferred in an attempt to minimize bias.

Biomarkers in orthodontics is a domain that needs to be thoroughly investigated. Biomarkers associated with OTM have been examined, while those associated with craniofacial growth and development are still under investigation. Current data are still scarce regarding whether and which GCF biomarkers are related to the growth phase (mainly pubertal growth spurts), while several investigations have been reported on GCF biomarkers (for inflammation, tissue damage, bone apposition, and resorption) in relation to OTM, as previously reported. Despite these investigations, the clinical applicability of the method is still limited and further data are needed to reach a conclusion. Future high-quality studies are warranted to elucidate the role of the main GCF biomarkers and how they can be used to enhance functional orthodontic and orthopedic treatment, optimize orthodontic force intensity, or prevent major tissue damage consequent to orthodontic treatment.

TMJ regeneration studies have been suggesting more and more that repair of the damaged area with the use of specific materials, with or without scaffolds, is becoming a possibility. Cartilage regeneration seems to be feasible even though the relevant studies have reported difficulties and limitations in clinical procedures. Tooth regeneration is also a promising domain with great potential. Unfortunately, the small number of available studies and the complexity of these procedures currently limit our knowledge in this field (Table 2).

A deeper knowledge of the mechanical and clinical stimuli that control tissue responses has been made possible by advances in bioengineering. According to tissue engineering, it is currently feasible to create living body parts from cells in a laboratory. Within the next few decades, tooth regeneration may be a possibility thanks to the foundations of experimental embryology, developmental and molecular biology, and the principles of biomimetics (the mimicking of biological processes). This is because the cellular, molecular, and developmental “rules” for tooth morphogenesis are rapidly being discovered [72,73,74].

As a result, in the not-too-distant future, it might be possible to restore missing tissue biologically using minimally invasive surgical techniques. The smile, midfacial height, and soft tissue draping of a patient can all be altered through the use of oral tissue engineering to replace lost osseous or dental components or repair orofacial abnormalities. It has been discovered that the amelogenin protein can self-assemble into nanospheres and it has regulatory effects on the bio-fabrication of enamel in mammalian teeth. Enamel biomineralization is negatively impacted by mutations that result in modifications to the amelogenin protein’s functional domains [75,76,77].

On the other hand, stem cells have been identified in dental pulp, and they consist of donor cells for various components of teeth. Furthermore, embryonic stem cells can now be cultured and even produced from adult cells by the nuclear transfer method.

In the biology of teeth, the discovery of dental epithelial stem cells in continuously growing teeth is a recent breakthrough [60]. These cells originate near the apical end of a tooth, which has a histological structure that is often specialized for maintaining adult stem cells and creating a variety of progenitor cells that produce dental tissues. Additionally, platelets secrete chemicals that aid in tissue healing and affect how vascular and other blood cells react during angiogenesis and inflammation. The platelet-derived growth factor (PDGF) has powerful effects on wound healing, including the rebuilding of the alveolar bone and cementum that support teeth (Table 2).

In this regard, donor cells, such as stem cells or cultivated differentiated cells, are seeded on a scaffold, replicating an extracellular matrix that has been configured suitably. The in-vitro system is supplemented with growth factors to promote the multiplication of stem cells. Then, the recipient receives the engineered structure [78].

We are now starting to understand, at the level of genes and molecules, how the development of dental tissues is regulated. At the moment, specific signal molecules have been identified which regulate the development of teeth and bones from progenitor cells [79]. Perhaps we will be able to grow new enamel, dentine, periodontal ligament, bone, or even whole new teeth for our patients in the future [80].

The opportunity for bioengineering to chart the course of tooth and bone regeneration is an exciting prospect, and it will improve the quality of life for patients in the decades to come. Therefore, an exciting area for research exists that will produce breakthrough knowledge to be published in the near future.

## 4. Conclusions

In conclusion, it comes as no surprise that biotechnology will change the way of thinking and practicing in the field of the craniofacial complex, offering further possibilities and modernizing the craniofacial clinical practice. However, there are still challenges to be overcome concerning the high cost, stability, and accessibility of these procedures, as human studies are still scarce and frequently have controversial results.

## Figures and Tables

**Table 1:** Biotechnological applications used for regeneration in the cranio-dento-facial complex. CLP: cleft lip and palate; TMJ: temporomandibular disorder.

	Stem Cells	Scaffolds	3D Printing	Natural or Synthetic Polymers
Craniofacial defects	+	+	+	
CLP	+	+	+	
TMJ regeneration	+	+		+
Tooth tissue regeneration	+			

**Table 2:** Applications of biotechnology for regeneration in the craniofacial complex.

Craniofacial Component	Application
Bone Regeneration	MSCs mediate and enhance bone regeneration. They are suitable cells for craniofacial bone repair. BMSCs contribute to the healing of cranial lesions. Stem cells from teeth and distraction osteogenesis are promising factors for face reconstruction. Composite scaffolds of demineralized bone material and calcium phosphate loaded with MSCs result in bone regeneration in cleft areas.
Temporomandibular Joint (TMJ) Regeneration	Scaffolds from natural and/or synthetic polymers loaded with MSCs in the TMJ area enable the differentiation of encapsulated MSCs into distinct fibrocartilage formations, and as a result, various types of cartilage tissues are formed.
Tooth Regeneration	Dental mesenchymal stem cells are suitable as dental pulp stem cells, and periodontal ligament stem cells and exfoliated deciduous teeth stem cells enhance pulp and periodontal regeneration.

## Data Availability

Not applicable.

## Abbreviations

CLP: cleft lip and palate; TMJ: temporomandibular disorder; MSC: mesenchymal stem cells; SCs: stem cells; DO: distraction osteogenesis; BMD: bone mineral density; OTM: orthodontic tooth movement.
